# Assessments of Impacts of Nitrogen Deposition on Beech Forests: Results from the Pan-European Intensive Monitoring Programme

**DOI:** 10.1100/tsw.2001.325

**Published:** 2001-11-22

**Authors:** Johannes Eichhorn, Thomas Haussmann, Uwe Paar, Gert Jan Reinds, Wim de Vries

**Affiliations:** Hessen Forst FIV, D-34346 Hann, Münden, Germany; BMVEL/ICP Forests, P.O. Box 150270, D-53107, Bonn, Germany; FIMCI Forest Intensive Monitoring Co-ordinating Institute, P.O.Box 47, NL-6700 AC Wageningen, The Netherlands; Alterra Green World Research, P.O. Box 47, 6700 AA Wageningen, The Netherlands

**Keywords:** beech (Fagus sylvatica), nitrogen, air pollution, N deposition, N leaching, N budget, N foliage, roots, Intensive Monitoring Programme, ICP Forests

## Abstract

The article reviews effects of nitrogen (N) deposition on beech forest ecosystems in Europe. On the basis of beech plots of the Pan-European Monitoring Programme of ICP Forests and the EU, the deposition of N compounds as well as input-output budgets are listed and compared with studies in North America. The authors also discuss the critical threshold for N leaching. At present, N is leached in 10% of the plots evaluated. An in-depth evaluation of a beech plot in central Germany is presented. The high N leaching results in a considerable increase (four times higher N content in 2000 compared to 1965) in the export of nitrate from the beech forests from a nearby source. Finally, ecophysiological indicators (N content in beech leaves, fine root system, N content, root/shoot ratios) are discussed as a result of high N input.

### INTRODUCTION

#### General Considerations

##### *Nitrogen Nutrition of Plants*

Nitrogen (N) holds an exceptional position among plant nutrients. On the one hand, N is the element for which plants have the highest demand. The availability of N compounds controls many aspects of biogeochemical processes and exerts a strong influence on net primary production in terrestrial ecosystems. On the other hand, it exhibits the special feature that the N content of the bedrock minerals is negligible[1] and thus is of no importance for plant nutrition. Because of the lack of N in minerals, all the N present in a forest floor originates from the atmosphere.

In many ecosystems, the limited input of N was a restrictive factor for plant growth[2] until the beginning of the industrial revolution[3,4,5,6,7]. Most ecosystems evolved under N-deficient environmental conditions.

##### *Definition of N Saturation in Forest Ecosystems*

A permanent enrichment of nutrients can lead to saturation phenomena in ecosystems. Cole[8] and Van Miegrot[9,10] define an ecosystem to be in the state of N saturation when atmospheric input and N mineralisation together exceed the retention capacity of a given ecosystem and lead to a permanent leaching of nitrate ($NO_3$). This definition relates to the biological concept of critical loads. According to those authors, for each given ecosystem, there exists a defined level of maximal tolerable N input. If the N input is below this limit, no excess $NO_3$ will be exported[11]. A similar definition has been given by Aber[12], defining N saturation as a shift in ecosystem dynamics from no leakage of inorganic N to its leakage[13].

### Background of the Pan-European Forest Monitoring Programme

Since the mid-1980s, forest condition has been monitored as a reaction to the growing concern that air pollution affects European forests. The UN/ECE has mandated coordination of these activities for ICP Forests in close cooperation with the EU (Regulation EEC No. 3528/86)[14].

In 1994, the Pan-European Forest Monitoring Programme was established within the ICP Forests and EU programme for the protection of forests from atmospheric pollution[15,16] with the aims to:

- Monitor the effects of anthropogenic (in particular air pollution) and natural stress factors on the condition and development of forest ecosystems in Europe (Systematic Grid, Level I)
- Contribute to a better understanding of cause-effect relationships in forest ecosystems functioning in various parts of Europe (Intensive Monitoring Programme, Level II)

Recently, the goals of the Intensive Monitoring Programme have been widened to include the topics of biodiversity, climate change, and the development and monitoring of criteria for sustainable forest management. The ICP Forests and EU programme provide a unique opportunity to monitor the effects of N deposition in forest ecosystems as important stress factors. Deposition, air concentration, and meteorological conditions are assessed, and biotic reactions of the forest ecosystems are observed.

### Aim of the Article

This contribution will summarise N data for beech plots included in the Intensive Monitoring Programme. It reports on impacts of N deposition on beech forests in Europe. Is there a serious N deposition affecting European beech forests? What is the impact of N input on tree nutrition and $NO_3$ output of beech stands in Europe?

## METHODS

### Beech Plots in the Intensive Monitoring Programme

The Intensive Monitoring Programme includes a total of 862 plots from 30 participating European countries. This article focuses on the 49 plots with European beech (*Fagus sylvatica*) as the dominant tree species.

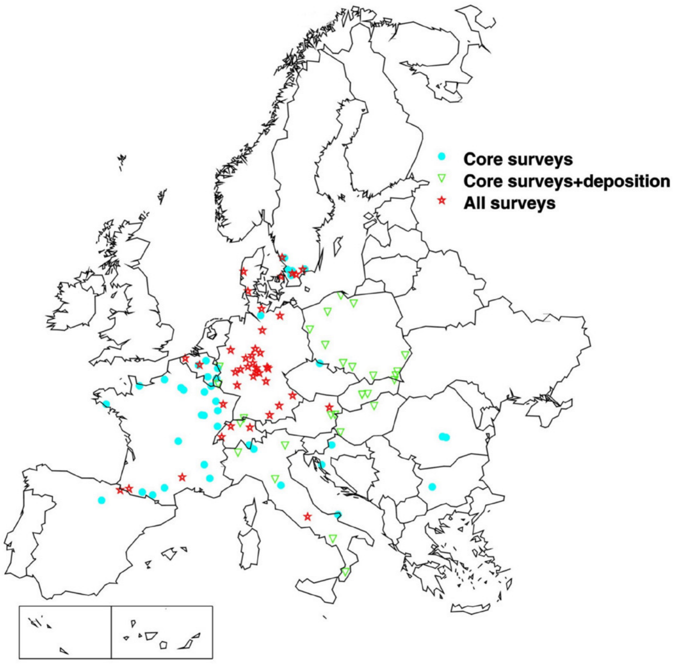

**FIGURE 1.** Surveys performed at the Intensive Monitoring Program plots with *F. sylvatica* as dominant species.

Fig. 1 shows the location of the beech plots and the different types of surveys that are carried out at these plots. On all plots, the crown condition, soil, foliage, and increment are monitored (core survey), whereas additional measurements are optional and applied on only selected plots.

### Budget Calculations

Hydrological fluxes were measured and calculated according to the methods described by De Vries[17]. A canopy exchange model developed by Ulrich[18,19] and extended by Draaijers[20] was used. Element outputs from the forest ecosystem were calculated from data of the soil solution composition at a depth below the root zone and simulated soil water fluxes during those periods. Element retention or release was assessed from the difference between the leaching from the bottom of the root zone and the element input from the atmosphere. Results are presented for the period 1995–1998.

## RESULTS

### Deposition of N Compounds

Ammonium ($NH_4$) throughfall deposition is measured for 49 plots of European beech plots. $NH_4$ throughfall ranges from 0.15 to 1.4 kmol$_c$ ha$^{-1}$ year$^{-1}$ (average: 0.53; Fig. 2). $NO_3$ varies from 0.2 to 1.6 kmol$_c$ ha$^{-1}$ year$^{-1}$ (average: 0.52). The average summarised N throughfall deposition is 1.1 kmol$_c$ ha$^{-1}$ year$^{-1}$.

In general, the annual average input ratio $NH_4/NO_3$ (bulk deposition as well as throughfall) varies between 1.1 and 1.2. However, plots with specific high $NO_3$ input values (bulk: >0.6 N kmol$_c$ ha$^{-1}$ year$^{-1}$; throughfall: >1.0) or exceptional $NH_4$ input sometimes do not show corresponding values for the other N component. This can probably be explained by the location of these sites close to polluting sources that emit large amounts of either $NH_3$ or $NO_3$ (respectively $NO_x$).

Fig. 2 shows the correlation between bulk deposition and throughfall of $NH_4$ deposition. Up to an $NH_4$ bulk deposition of 0.55 kmol$_c$ ha$^{-1}$ year$^{-1}$, there is a very clear correlation between bulk and throughfall; above this value, the correlation is less obvious.

A similar relation was found between bulk and throughfall of $NO_3$. In both cases, the slope of the regression functions has a value close to 1.0.

### Input-Output Budgets

The total N deposition of European beech plots ranges from 0.59 to 2.1 kmol$_c$ ha$^{-1}$ year$^{-1}$ (average: 1.36 kmol$_c$ ha$^{-1}$ year$^{-1}$; Fig. 3). The $NH_4/NO_3$ ratio for the total deposition is 1.19. These values confirm the findings of other long-term *F. sylvatica* case studies in Germany[21] (1995: average total deposition N: 1.55 kmol$_c$ ha$^{-1}$ year$^{-1}$). Total N input is generally higher in Central and Western Europe than in Northern and Southern Europe.

The Integrated Forest Study in the Eastern U.S.[22] revealed, however, that the majority of the monitored stands (80%) had an N input (total N deposition) that was lower than 1 kmol$_c$ ha$^{-1}$

**TABLE 1**
**Regression Coefficients for $NH_4$ Throughfall (NH4THROUGH) vs. $NH_4$ Bulk Deposition (NH4BULK) in *F. sylvatica***

**Regression coefficients**
**NH4THROUGH vs. NH4BULK**

| | coefficient | std.err. | std.coeff. | t | P |
|---|---|---|---|---|---|
| intercept | .137 | .080 | .137 | 1.715 | .0930 |
| NH4BULK | .977 | .186 | .608 | 5.247 | <.0001 |

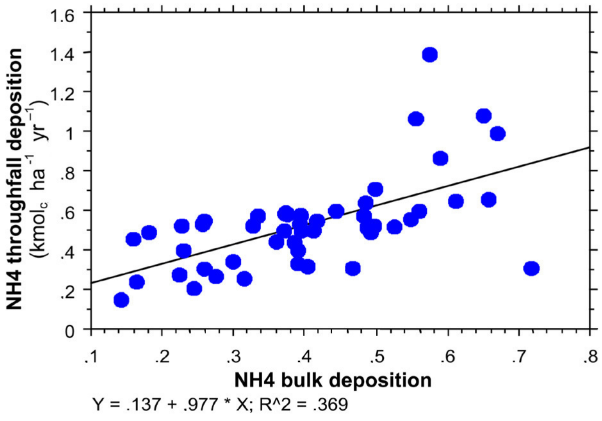

**FIGURE 2.** $NH_4$ throughfall vs. $NH_4$ bulk deposition in *F. sylvatica.*

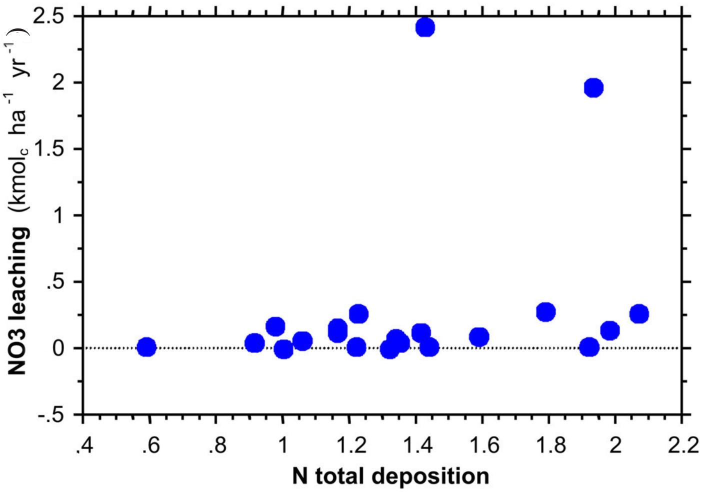

**FIGURE 3.** N total deposition vs. N leaching (*F. sylvatica*, Intensive Monitoring Programme).

year$^{-1}$ (average: 0.8 kmol$_c$ ha$^{-1}$ year$^{-1}$). The lower deposition load in these forests represents N loads that are closer to conditions without anthropogenic impact[5].

Element leaching fluxes show that N leaching greatly varies for different beech ecosystems. For the Intensive Monitoring Programme beech plots with soil solution measurements, $NO_3$ output varies from 0.05 to 2.4 kmol$_c$ ha$^{-1}$ year$^{-1}$ (Fig. 3). The frequency distribution may be interpreted as a bimodal function. A total of 91% (20/22) exhibit negligible N leaching fluxes (below 0.5 kmol$_c$ ha$^{-1}$ year$^{-1}$). Only 9% (2/22) reveal a $NO_3$ leaching up to 2.42 kmol$_c$ ha$^{-1}$ year$^{-1}$.

The average leaching of 0.293 kmol$_c$ ha$^{-1}$ year$^{-1}$ indicates that, as a rule, N is strongly retained in the ecosystems investigated, since the leaching is much lower than the N input.

Eichhorn[21] suggested that a total N deposition of 1 kmol$_c$ ha$^{-1}$ year$^{-1}$ and higher could be taken as an indicator for $NO_3$ leaching. Dise[23] and Gundersen[24] defined 0.7 kmol$_c$ ha$^{-1}$ year$^{-1}$ as the critical threshold. Below this limit, leaching of N seems to be negligible. At N throughfall inputs above 0.7 kmol$_c$ ha$^{-1}$ year$^{-1}$, leaching of N can be expected to be elevated. However, no positive regression or a certain N input threshold for increased $NO_3$ leaching from the soil can be deducted from the data set of the beech plots.

At two sites, N leaching is considerable (above 0.7 kmol$_c$ ha$^{-1}$ year$^{-1}$) whereas the N input is moderate, indicating the occurrence of a disturbed N cycle[25,26]. One of these plots will be described in more detail later in this article.

### Total N Deposition and N Content in the Foliage

Fig. 4 shows a clear impact of N deposition on the N status of beech leaves. The high deposition rates of $NH_4$ lead to a high percentage of forest sites with a good availability of N in leaves. This is reflected by the high N content of the foliage. A comparison of different N components shows that $NH_4$ deposition has the strongest influence on the N content in leaves. However, there is no statistical correlation between $NO_3$ total throughfall deposition and N content of foliage (Table 1). This can be explained by the preference of beech for $NH_4$ as N nutrition[27].

## RESPONSE OF AN N-SATURATED BEECH FOREST ECOSYSTEM (INTENSIVE MONITORING LEVEL II, PLOT ZIERENBERG)

### Introduction

The Zierenberg Forest is located in northern Hesse on the hillside Kleiner Gudenberg. This is close to the town of Zierenberg, northwest of Kassel. It is a site that has been intensively studied for N saturation and the ecosystem response. It has a comparatively high N store of about 790 kmol N ha$^{-1}$ in its forest floor.

The plant community is basically a *Galium odoratum-Fagetum circetosum*[28]. At present, it carries a 156-year-old beech forest with a few ash and maple trees. The trees have a mean height of 36 m and an average diameter of 58.3 cm and represent a high productivity class (8.6 m$^3$ ha$^{-1}$ year$^{-1}$ annual increment of wood above 7 cm in diameter; standing crop 530 m$^3$). The ground vegetation is dominated by nitrophile plants such as stinging nettle (*Urtica dioica*).

The stand is situated on a basalt cone over shelly lime. The soil chemistry data[29,30] are given in Table 3.

The Zierenberg results are compared to intensive monitoring plots of the "Forest Ecosystem Study Hesse" (for site description, see[21]).

### N Status of Zierenberg

#### *N Budget*

At the Zierenberg site, total N deposition varies from 1.5 to 2.0 kmol$_c$ ha$^{-1}$ year$^{-1}$ (Fig. 5). An average of 0.82 kmol$_c$ ha$^{-1}$

**TABLE 2**
**Regression Coefficient N Content Foliage (NFOLIAGE) vs. $NH_4$ Total Deposition (NH4DEPTOTAL) in *F. sylvatica***

**Regression coefficient**
**NFOLIAGE vs. NH4DEPTOTAL**

| | coefficient | std.err | std.coeff. | t | P |
|---|---|---|---|---|---|
| intercept | 20.131 | .849 | 20.131 | 23.718 | <.0001 |
| NH4DEPTOTAL | 3.851 | 1.034 | .631 | 3.725 | .0013 |

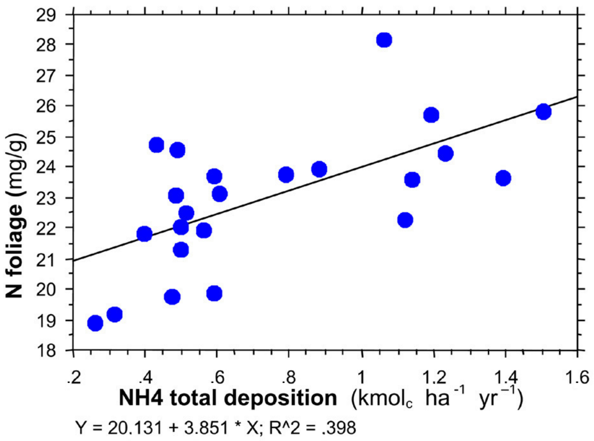

**FIGURE 4.** N content foliage (*F. sylvatica*; vegetation period sampling) vs. $NH_4$ total deposition.

**TABLE 3**
**Zierenberg: Soil Chemistry, Mineral Soil (0 to 80 cm)**

| | Soil Depth (cm) | 0–5 | 5–10 | 10–20 | 20–40 | 40–80 |
|---|---|---|---|---|---|---|
| | Ake mmol eq/kg | 141 | 109 | 101 | 101 | 147 |
| | Base saturation % | 96 | 93 | 92 | 97 | 99.5 |
| | $pH_{KCl}$ | 4.7 | 4.5 | 4.6 | 5.8 | 5.25 |
| | $pH_{H_2O}$ | 5.3 | 5.25 | 5.3 | 5.6 | 6.05 |
| Total content (kg/ha) | Org. C | 115000 | | | | |
| | N | 10400 | | | | |
| Exchangeable Content (kg/ha) | Ca | 10700 | | | | |
| | Mg | 4000 | | | | |

year$^{-1}$ $NH_4$ represents 48% of the total N input (1.71 kmol$_c$ ha$^{-1}$ year$^{-1}$). No temporal trend is visible. The output values show a remarkable variability. During the last years, $NO_3$ output exceeded 2 kmol$_c$ ha$^{-1}$ year$^{-1}$. In 1998, a maximum of 2.42 kmol$_c$ ha$^{-1}$ year$^{-1}$ was reached.

Fixation of atmospheric N[6], N accumulated in tree increment, and denitrification from field experiments at a neighbouring site were measured. The actual output is higher than the average value. The assumed N fixation values were given by the authors for forests with no anthropogenic N input.

The soil budget derived from measurements indicates an average loss of 1.1 kmol$_c$ ha$^{-1}$ year$^{-1}$ N per year (Table 4).

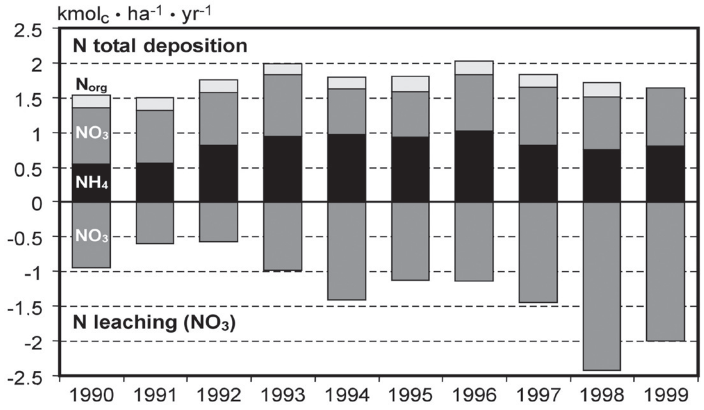

**FIGURE 5.** N budget Level II-plot Zierenberg for 1990–1999.

**TABLE 4**
**N Content Foliage (*F. sylvatica*; Vegetation Period Sampling) vs. $NO_3$ Total Deposition**

| Regression Coefficient NFOLIAGE vs. | Coefficient | std. err | std. coeff. | t | P |
|---|---|---|---|---|---|
| Intercept | 22.897 | 1.816 | 22.897 | 12.606 | <.0001 |
| NO3DEPTOTAL | .123 | 2.818 | .009 | .044 | .9657 |

**TABLE 5**
**Soil N Budget for Zierenberg (kmol$_c$ ha$^{-1}$ year$^{-1}$), Average for 1990–1999**

| | | | |
|---|---|---|---|
| **Input** | | | |
| **Total N Deposition** | **N Fixation** | | **Sum** |
| 1.71 | 0.36 | | 2.07 |
| **Output Soil** | | | |
| **Tree Increment** | **Nitrate Leaching** | **Denitrification** | |
| 1.56 | 1.26 | 0.36 | 3.18 |
| **Budget Soil** | | | |
| | | | –1.11 |

### *Nitrate Concentration in the Soil Solution at Level II Plot Zierenberg*

Figure 6 shows the temporal variability of $NO_3$ concentrations in two different soil depths. The mean (biweekly average from 1990 to 2000) nitrate concentration in 20-cm depth is 689 µmol$_c$ l$^{-1}$; the corresponding value for 60 cm is 773 µmol$_c$ l$^{-1}$. Lower $NO_3$ concentrations are found during the vegetation period where plants and micro-organisms probably use part of the N for nutrition purposes. During the nonvegetation period, maxima of nitrate concentrations appear.

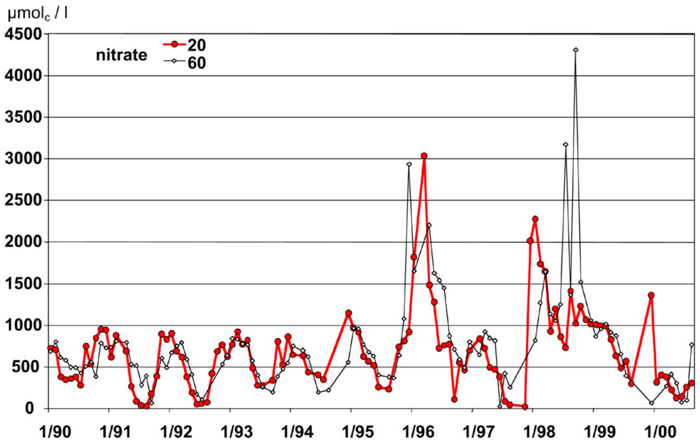

**FIGURE 6.** Nitrate concentrations for Zierenberg (soil depth 20 and 60 cm).

### *Source Friedrichsaue*

The source Friedrichsaue integrates the watershed system of the Zierenberg forest area. The use of public water supply reflects rather well the overall export of ions from the site[31]. Fig. 7 indicates a considerable increase in the export of $NO_3$ from the site. Whereas the N content in the 1960s was around 0.075 $mmol_{ieq}$ $l^{-1}$, the present $NO_3$ concentrations reach 0.35 $mmol_{ieq}$ $l^{-1}$. This is almost 4.7 times as much compared to 1965. The constant concentration of chloride ions indicates that the increase in $NO_3$ is not caused by a reduction of the watershedding of the source.

The source is used for public freshwater supply. This connects aspects of forest management and the needs of society. It is an example of the importance of a better understanding of processes that trigger nitrate leaching in forest areas.

### Physiological Response of the Stand

#### *Aluminum Loading of the Cell Walls of the Root Cortex*

The most sensitive tool for detecting a possible aluminum (Al) binding to plant cell walls is the method of ion localisation by analysis of the energy spectrum of backscattering x-rays in an electron microscope equipped with the EDAX system[32,33].

To interpret the findings of Zierenberg, a comparison with the nearby long-term monitoring stand Solling has been carried out.

Analysis of roots of beech using this method sampled at different times of the year both in the Solling, where the trees grow in very acidic soils, and from the Zierenberg site, where the overall pH of the soil solution is always significantly above 4.2 (an indicator of Al release from forest soils), revealed no significant differences with regard to the Al loading of the cell walls of the tree[34]. Results show that the roots from the Zierenberg site also have a high Al content in their root cell walls.

#### *Biomass and Root Distribution in Different Depths*

The root biomass of the beeches at the Zierenberg site was only 3/4 of the average root biomass of the other Hessian sites (ratio of the average root biomass for all soil depths: 430.5 mg/100 $cm^3$ for Zierenberg in relation to 589.4 mg 100 $cm^3$ = 73% for other Hessian sites). A more differential look at different root diameters showed a significantly smaller frac-

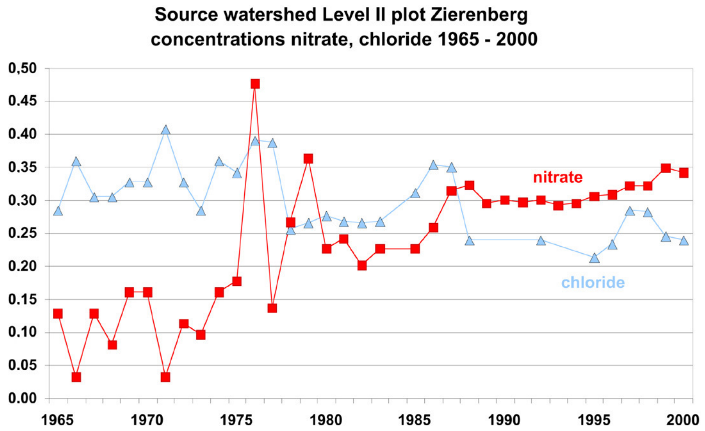

**FIGURE 7.** $NO_3$ and chloride concentrations for spring Friedrichsaue from 1965 to 2000.

tion of fine roots at the Zierenberg site. The generally smaller biomass of fine roots was strongly accompanied by mycorrhiza fungi. The root systems of old beeches in Zierenberg have a high abundance of mycorrhiza, whereas the biomass of fine roots is small. This enables optimal tree growth with an annual increment of 8.6 $m^3\,ha^{-1}$.

### C/N Ratio of the Organic Layer and Root Biomass

Statistical calculations show a positive correlation between the biomass of minor and fine roots and the C/N ratio of the soil. There is a significant positive correlation in the organic layer between the biomass of fine roots and the C/N (Fig. 8). On basaltic soil, beeches have fewer fine and minor roots than on sandstone.

### N and Root/Shoot Ratio of Old Beeches

To compare aboveground biomass with variables derived from root samples, an estimation of the biomass storage was carried out. In all beech monitoring sites, the shoot-root ratio is negatively correlated with the C/N ratio of the soil (Fig. 9). The more adverse the C/N ratio, the higher the root fraction of the biomass.

In particular, high concentrations of soluble and extractable N of the organic layer correlate positively with the shoot-root ratio of the beeches. It seems that beeches with a good availability of N generate a large aboveground biomass with comparatively few roots.

### CONCLUSIONS

The European Intensive Monitoring Programme of ICP Forests and EU yields reliable data on the N budget of European beech

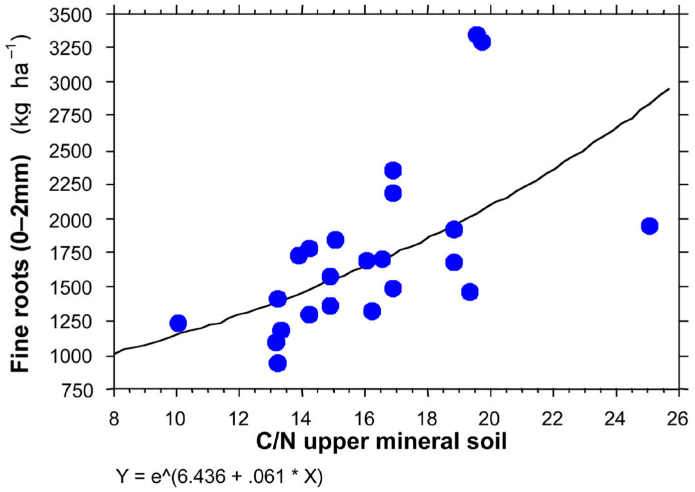

**FIGURE 8.** Fine root biomass (depth 0 to 50 cm) and C/N ratio in the upper mineral soil.

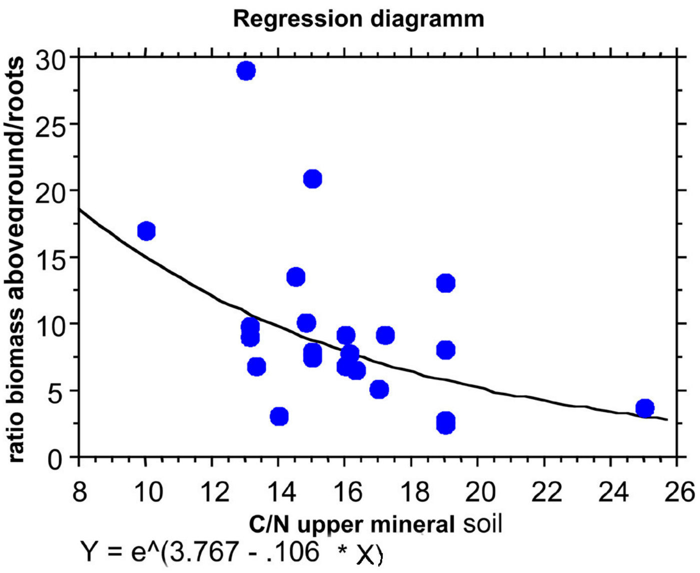

**FIGURE 9.** Beech stand (age above 100 years) ratio of total biomass aboveground / root vs. C/N upper mineral soil.

forests. The N input varies considerably, and the majority of these forests are still in the phase of N retention. About 10% of the studied plots, however, showed strong indications of N leaching. Excess N, especially in the form of ammonia, strongly influences the physiology of the trees.

Taking into account that the continuous N deposition in the majority of the forests is still considerably high, it can be expected that in the future, more forest ecosystems will reach the stage of N saturation and will reach N leaching, with all its detrimental consequences.

Intensive Monitoring Programme data provide quantitative information on sustainable forestry. These data should be considered when making local or regional forest management decisions.

The evaluation of root systems reveals important indicators of forest condition. Therefore, the inclusion of data of the root system in the Pan-European Monitoring Programme should be considered.

### ACKNOWLEDGEMENTS

This manuscript was substantially improved as a result of constructive comments provided by Joachim Gehrmann, Aloys Hüttermann, and Kare Venn.

The Institute for Forestry and Game Management, Laboratory of Forestry, Laboratory of Soil Science, University of Ghent, Forstliche Versuchsanstalt Rheinland-Pfalz, Trippstadt, Bayer. Landesanstalt für Wald und Forstwirtschaft München and Landesanstalt für Ökologie, Bodenordnung und Forsten, Recklinghausen supported the article by data contributions.

| | | |
|---|---|---|
| Received: | August | 8, 2001 |
| Revised: | October | 11, 2001 |
| Accepted: | October | 25, 2001 |
| Published: | November | 22, 2001 |
